# Supplementary figures and images for: Na+/K+- and Mg2+-ATPases and Their Interaction with AMPA, NMDA and D2 Dopamine Receptors in an Animal Model of Febrile Seizures
Source: Int J Mol Sci. 2022 Nov 24;23(23):14638. doi: 10.3390/ijms232314638 (PMC9737571; doi:10.3390/ijms232314638)

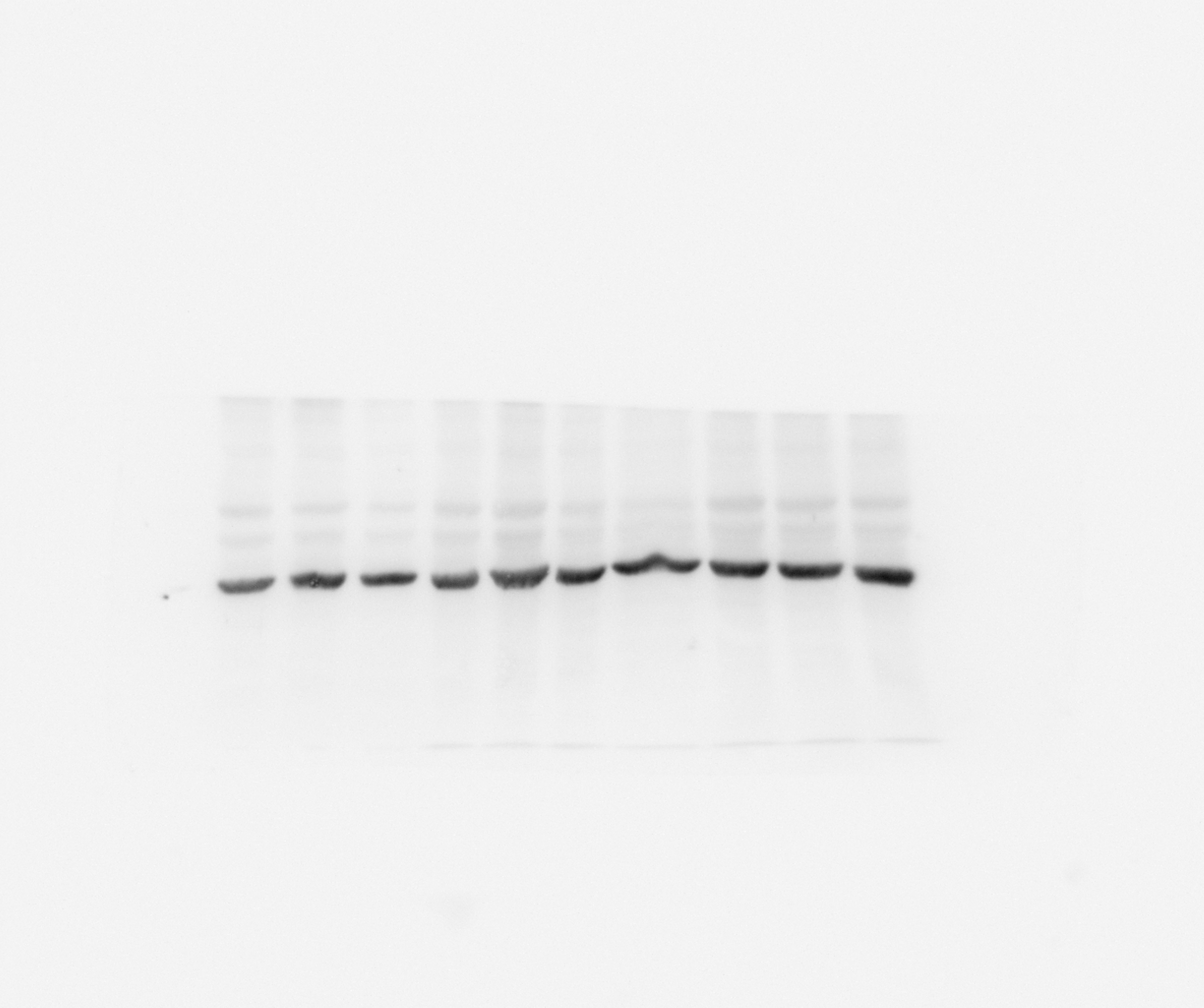

Supplement: Supplementary file 1 [file ijms-23-14638-s001.zip › Figure S1.tif]

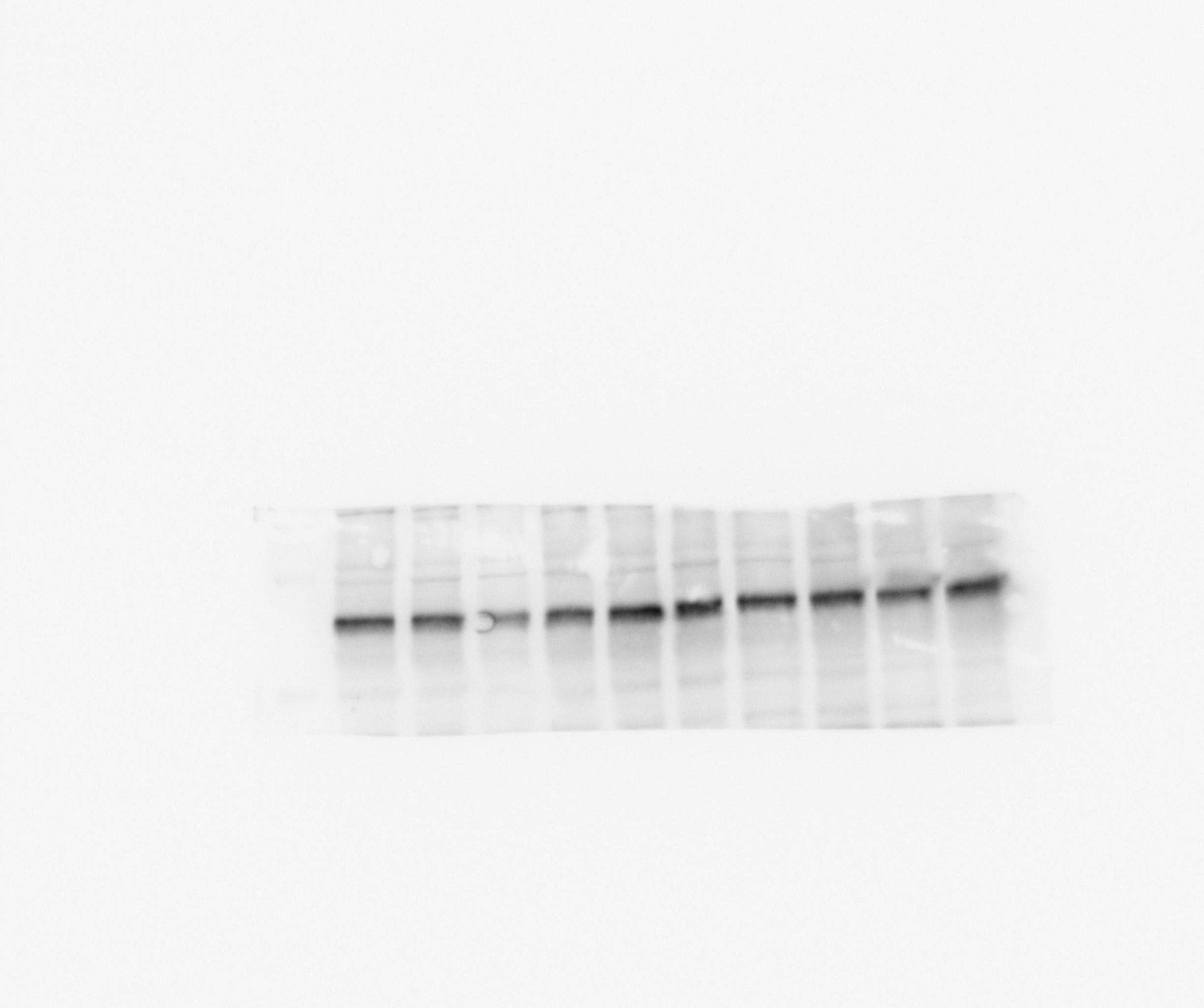

Supplement: Supplementary file 1 [file ijms-23-14638-s001.zip › Figure S10.tif]

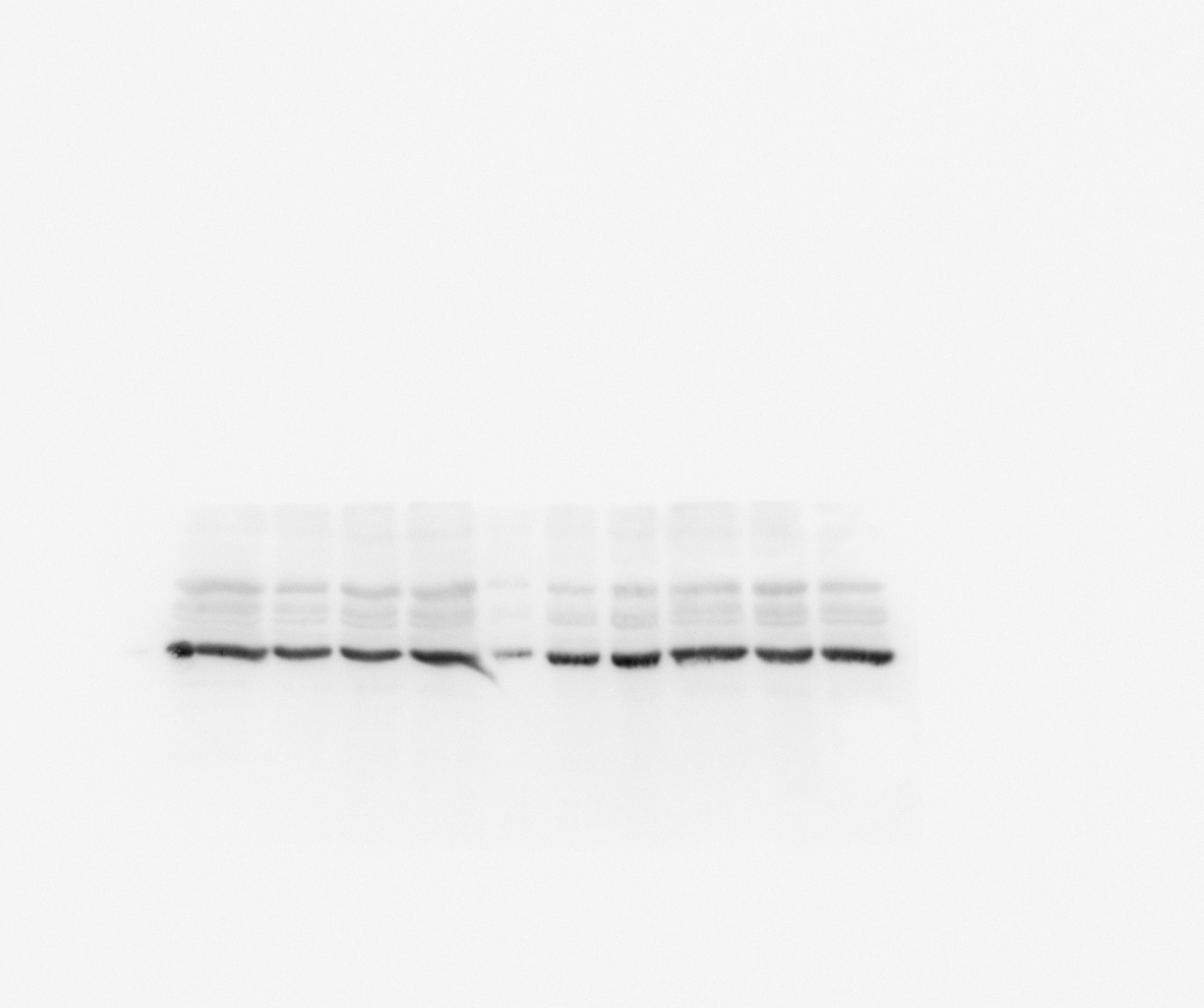

Supplement: Supplementary file 1 [file ijms-23-14638-s001.zip › Figure S11.tif]

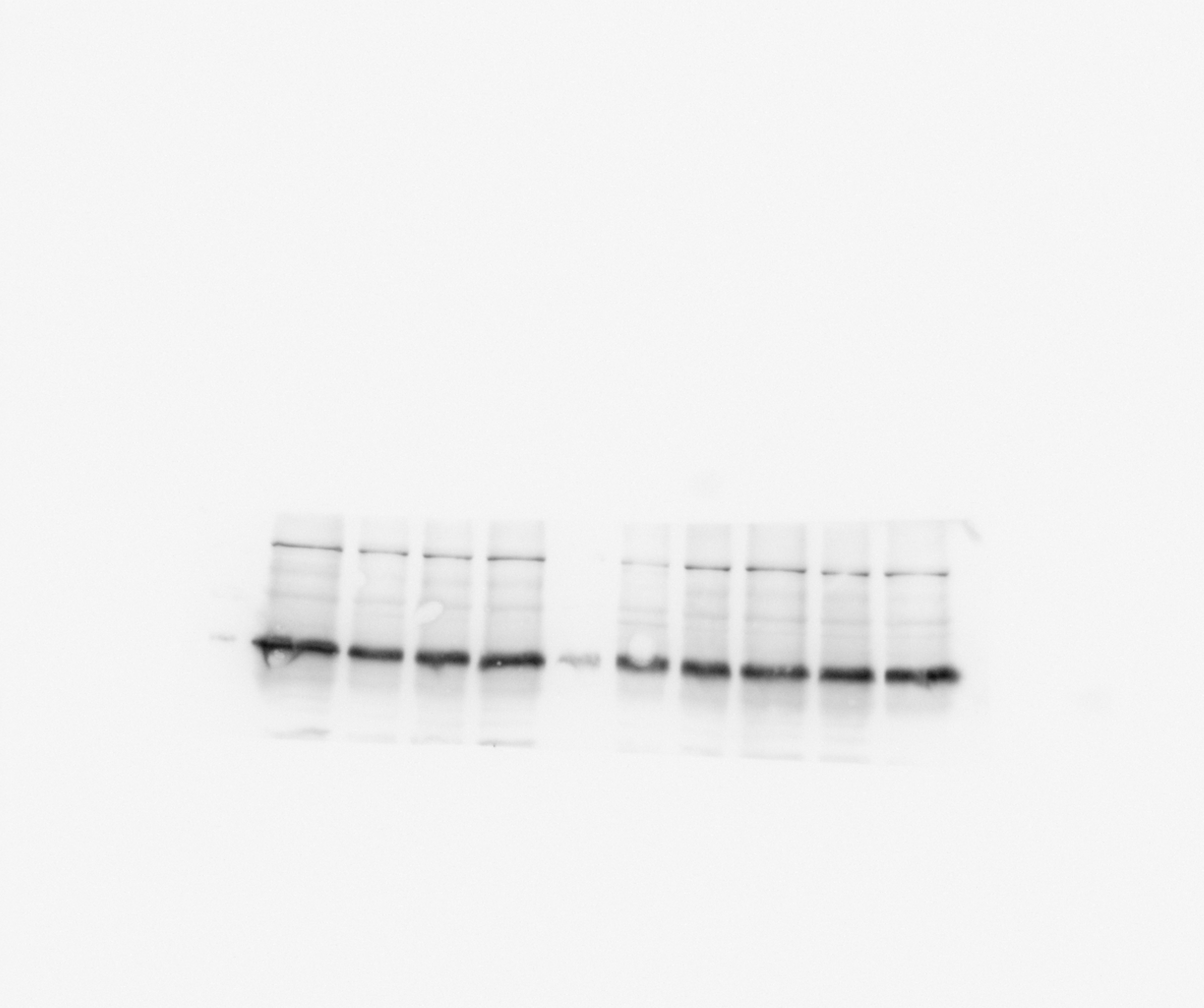

Supplement: Supplementary file 1 [file ijms-23-14638-s001.zip › Figure S12.tif]

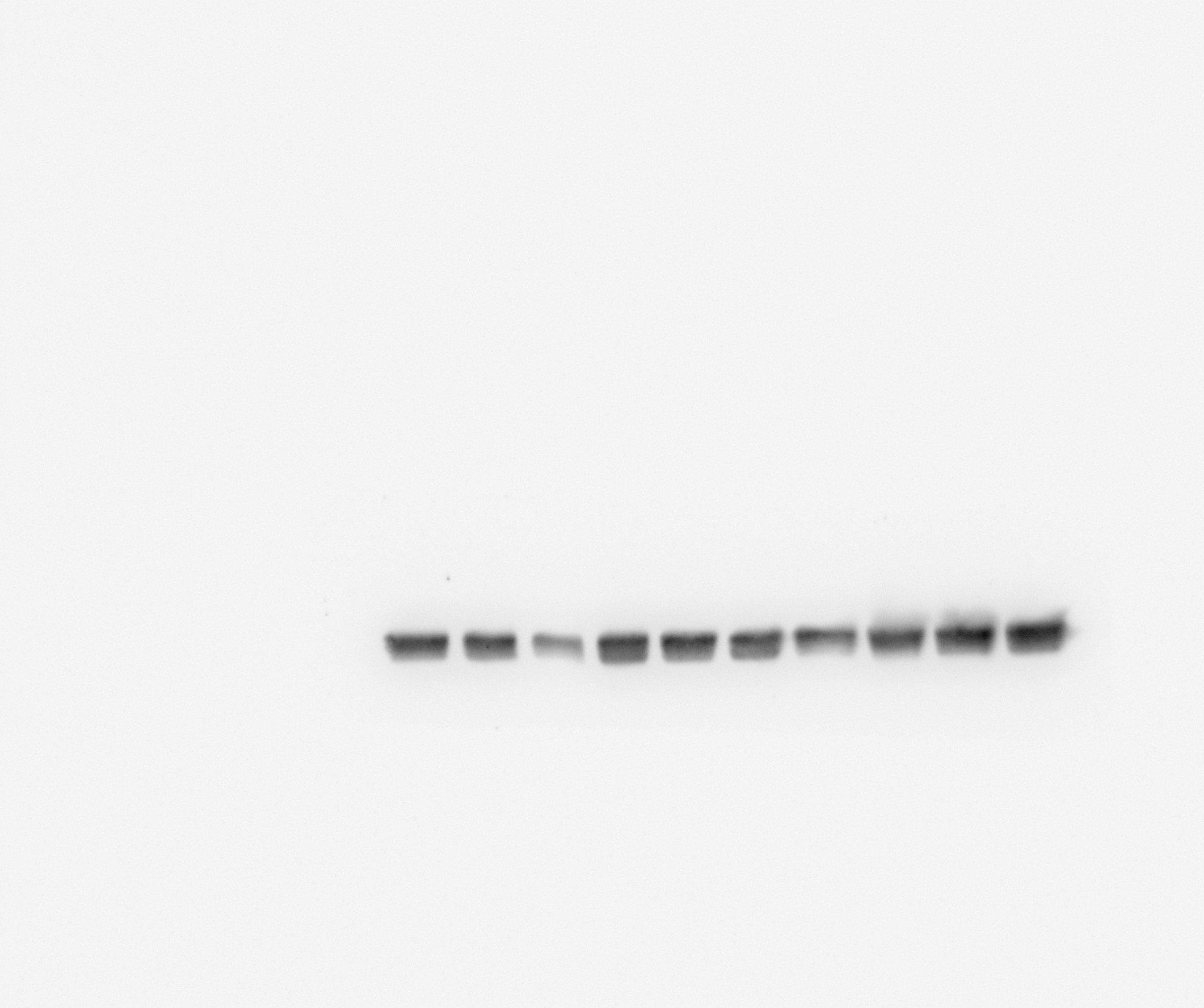

Supplement: Supplementary file 1 [file ijms-23-14638-s001.zip › Figure S13.tif]

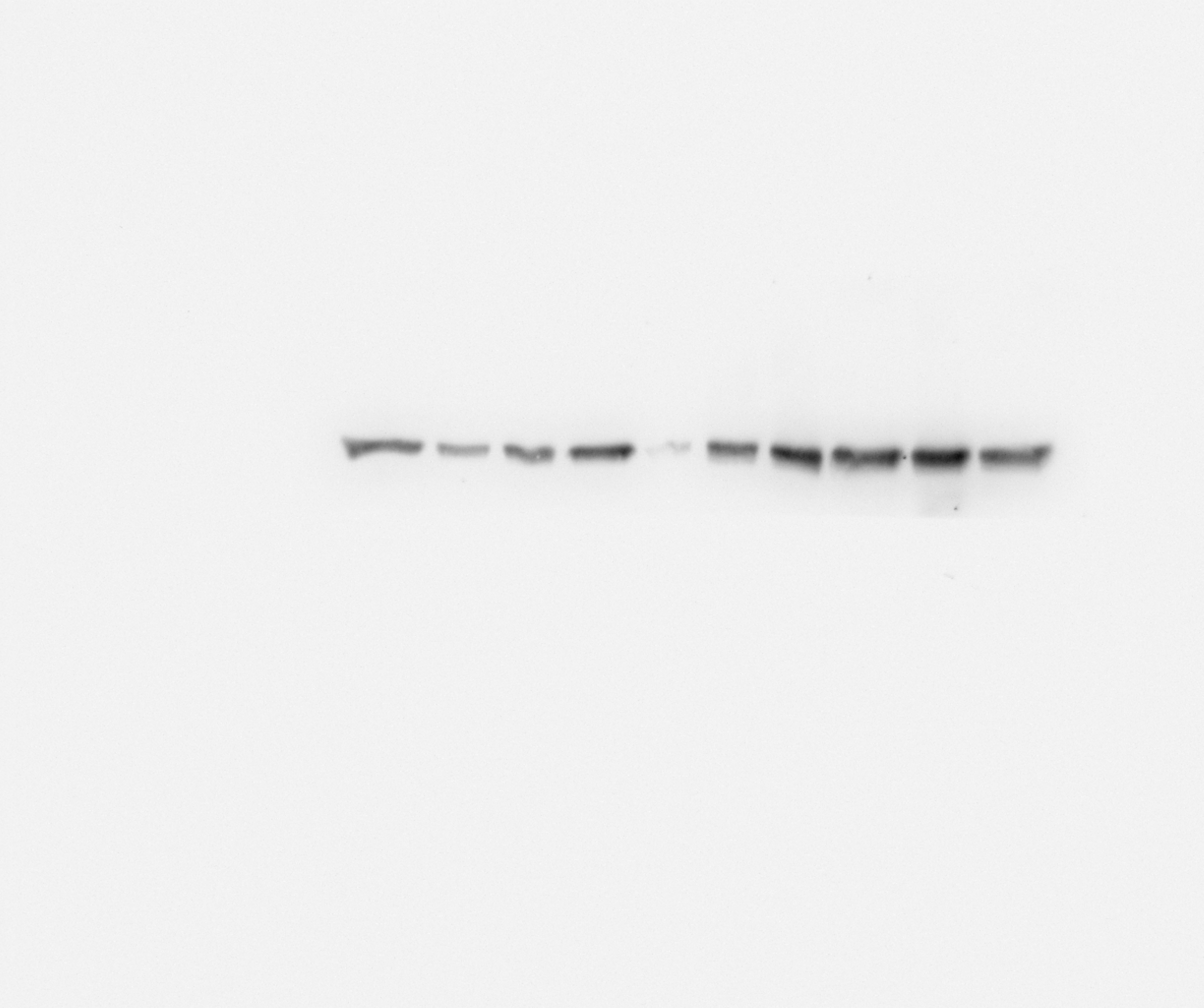

Supplement: Supplementary file 1 [file ijms-23-14638-s001.zip › Figure S14.tif]

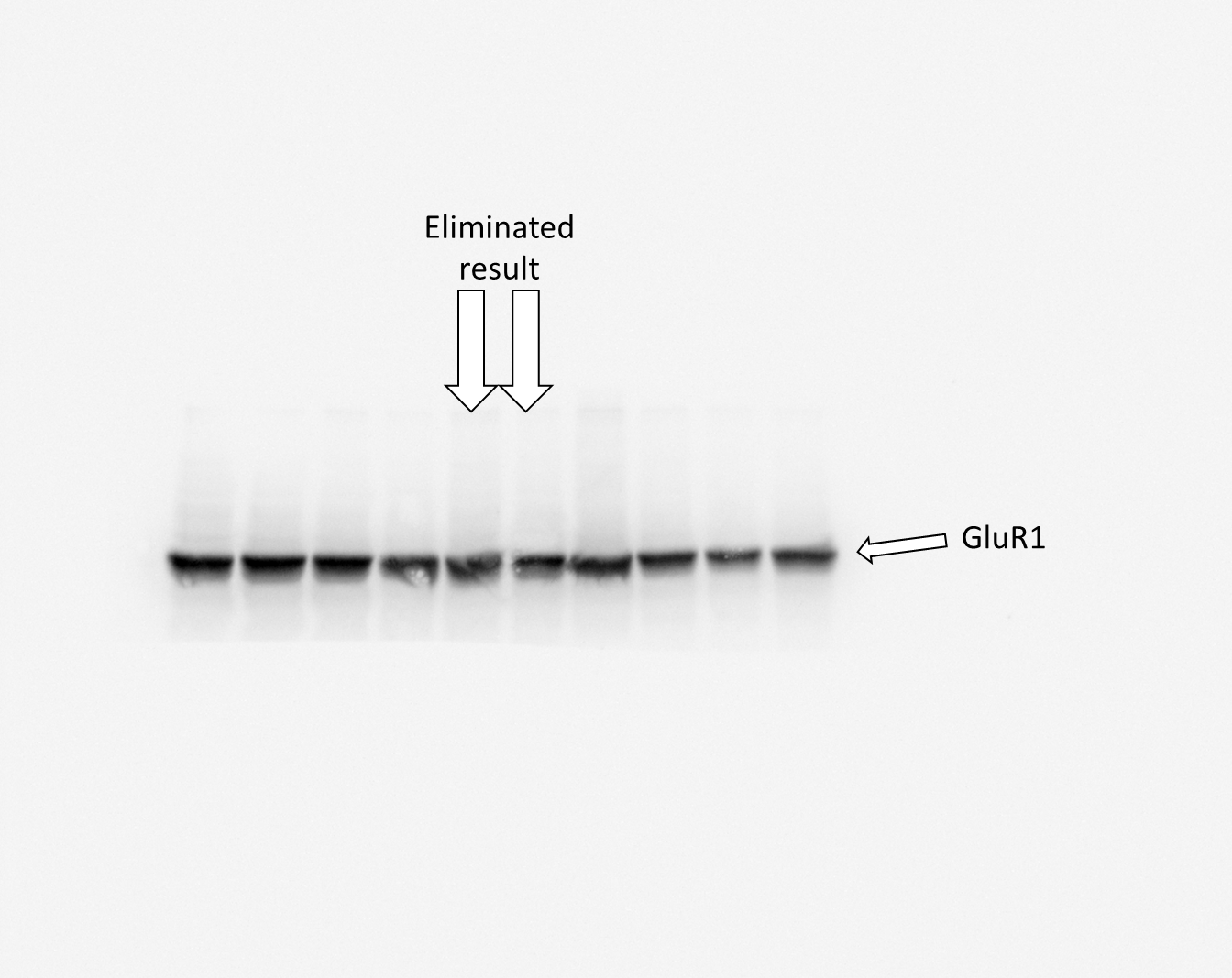

Supplement: Supplementary file 1 [file ijms-23-14638-s001.zip › Figure S15.tif]

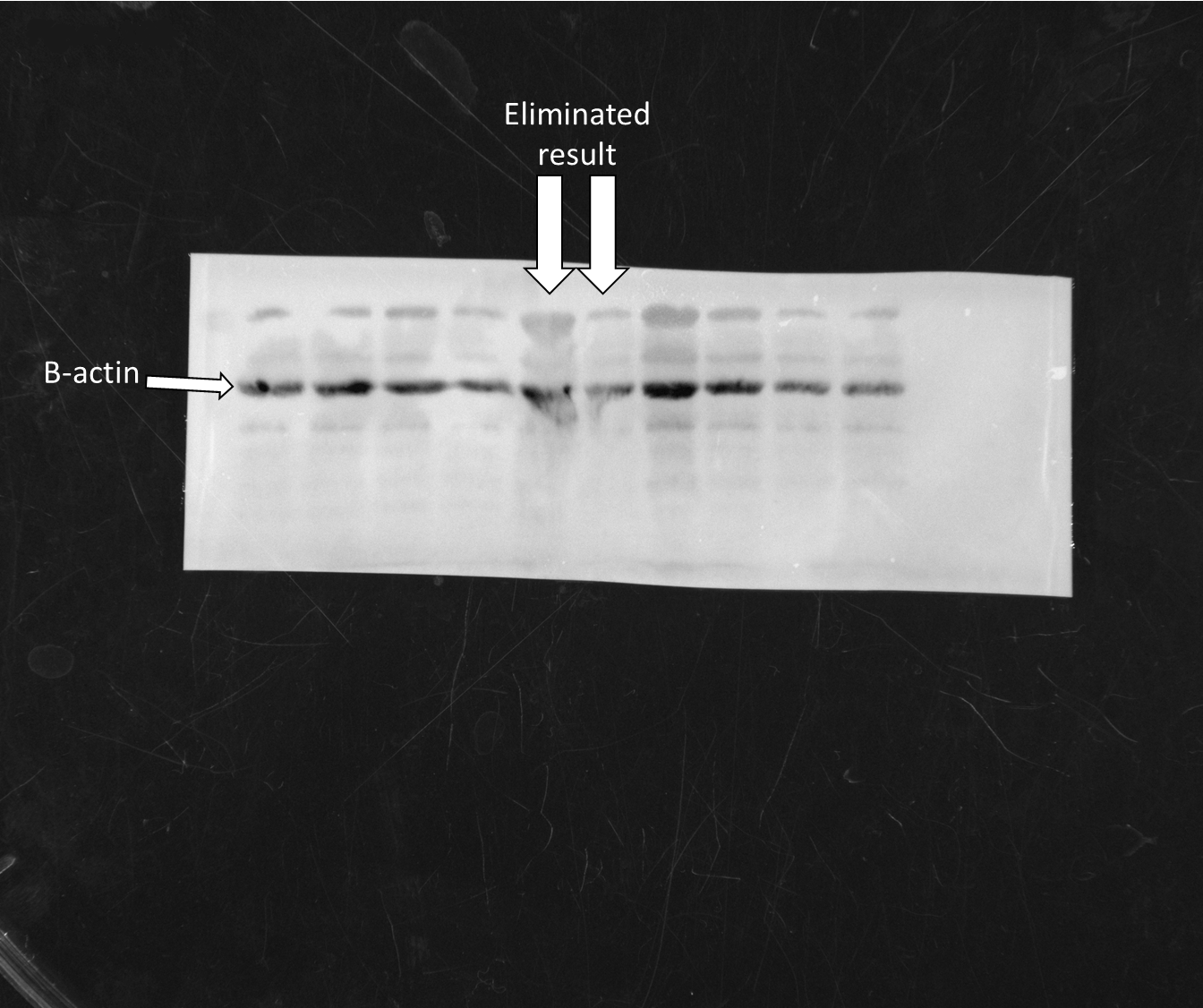

Supplement: Supplementary file 1 [file ijms-23-14638-s001.zip › Figure S16.tif]

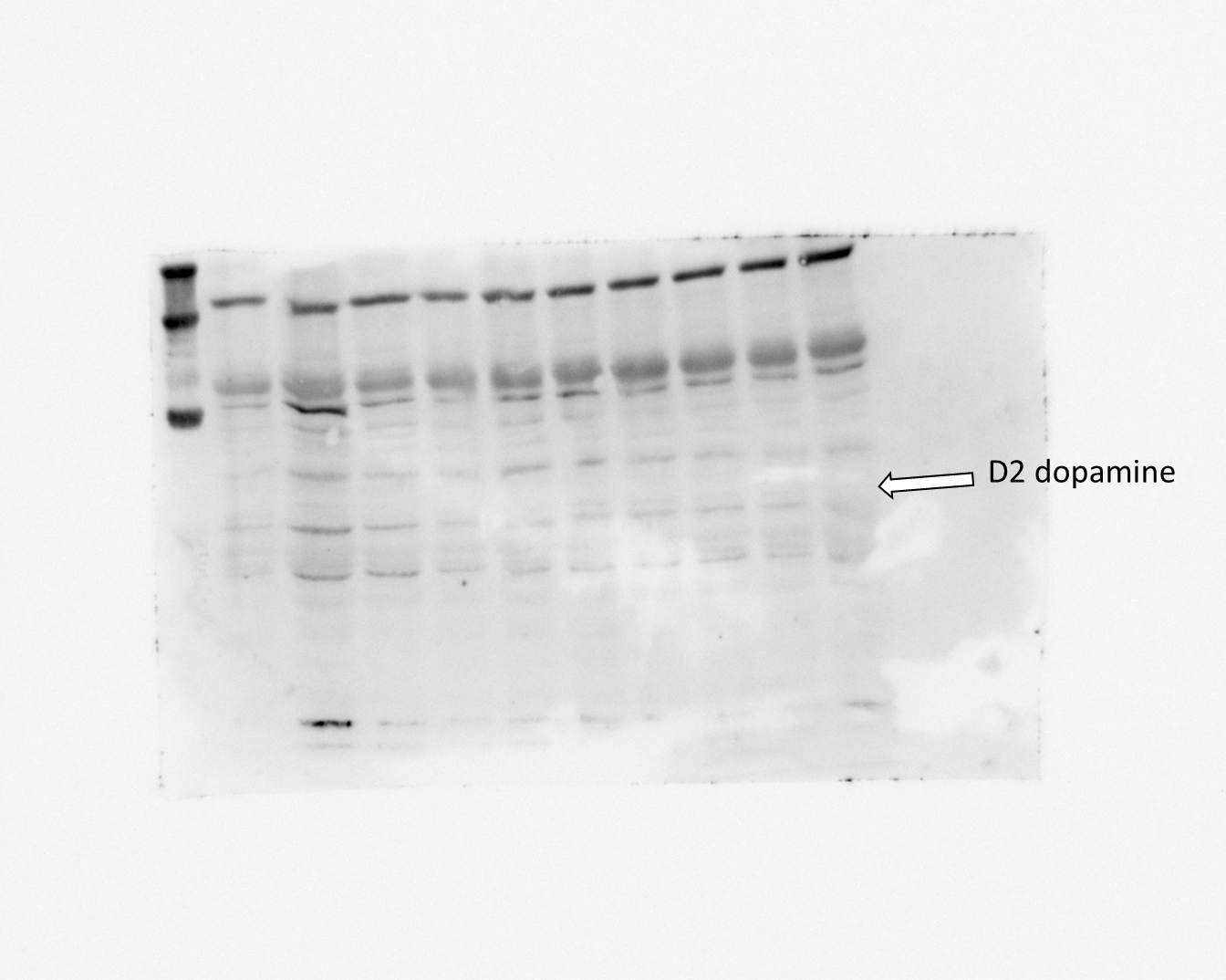

Supplement: Supplementary file 1 [file ijms-23-14638-s001.zip › Figure S17.tif]

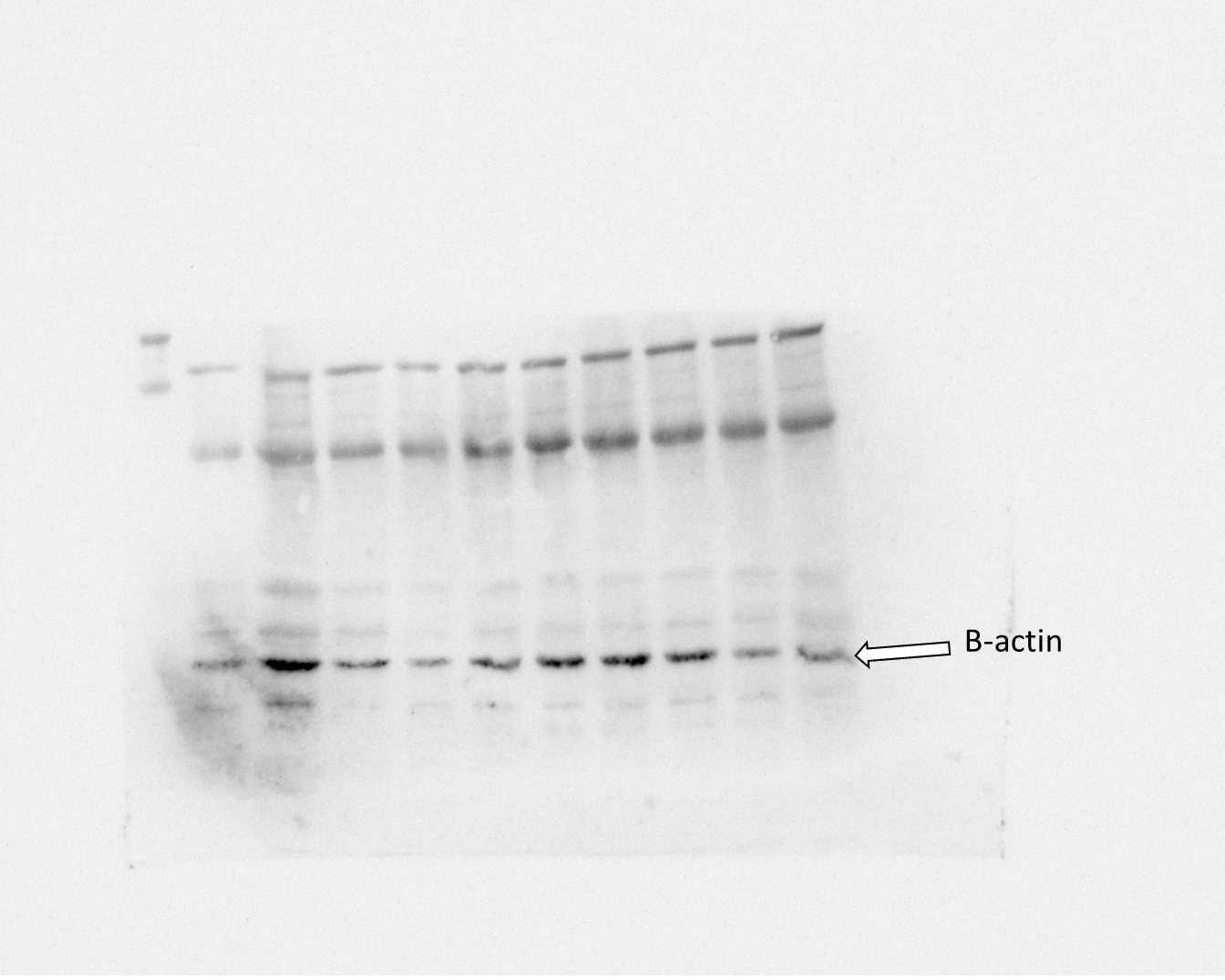

Supplement: Supplementary file 1 [file ijms-23-14638-s001.zip › Figure S18.tif]

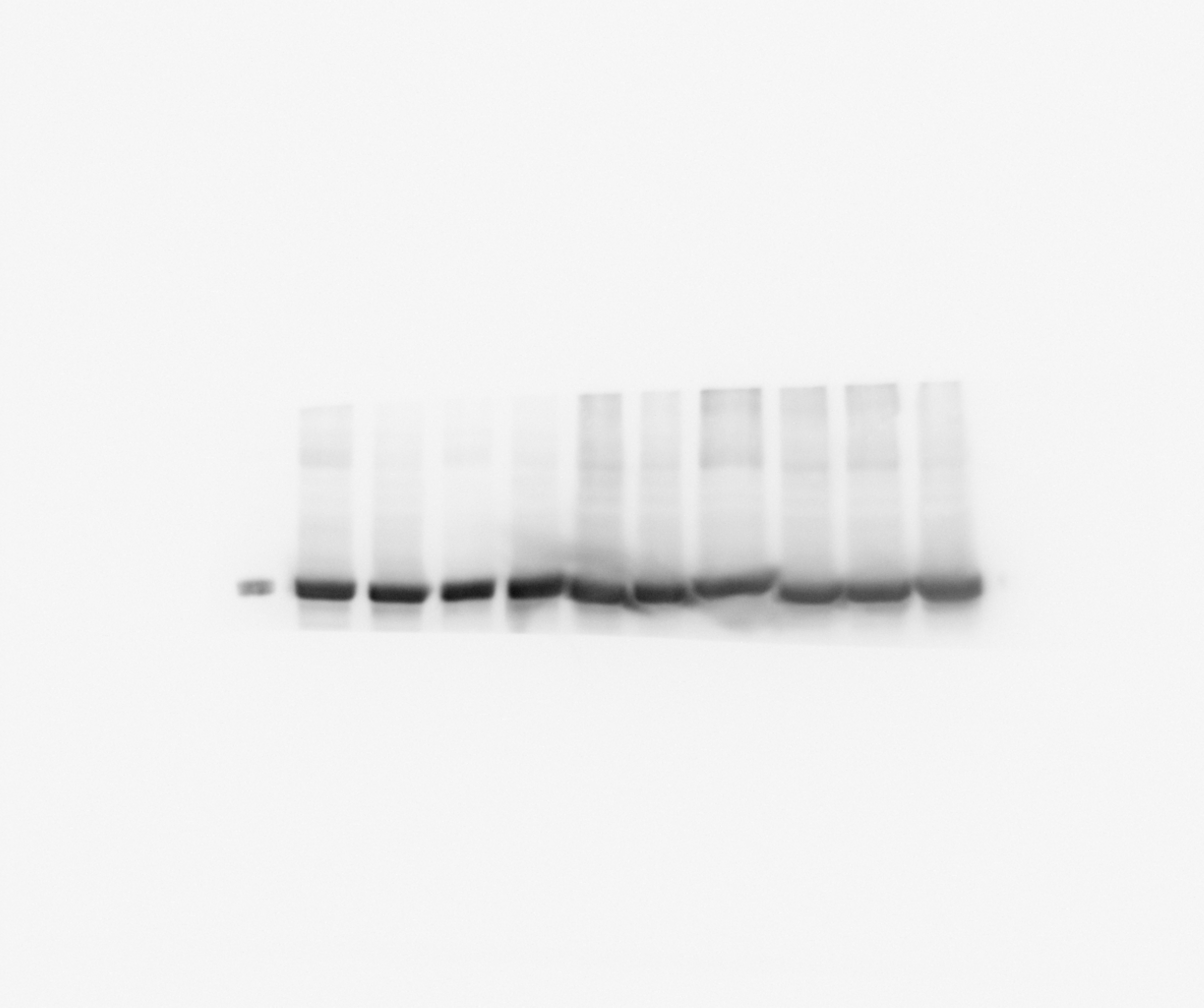

Supplement: Supplementary file 1 [file ijms-23-14638-s001.zip › Figure S2.tif]

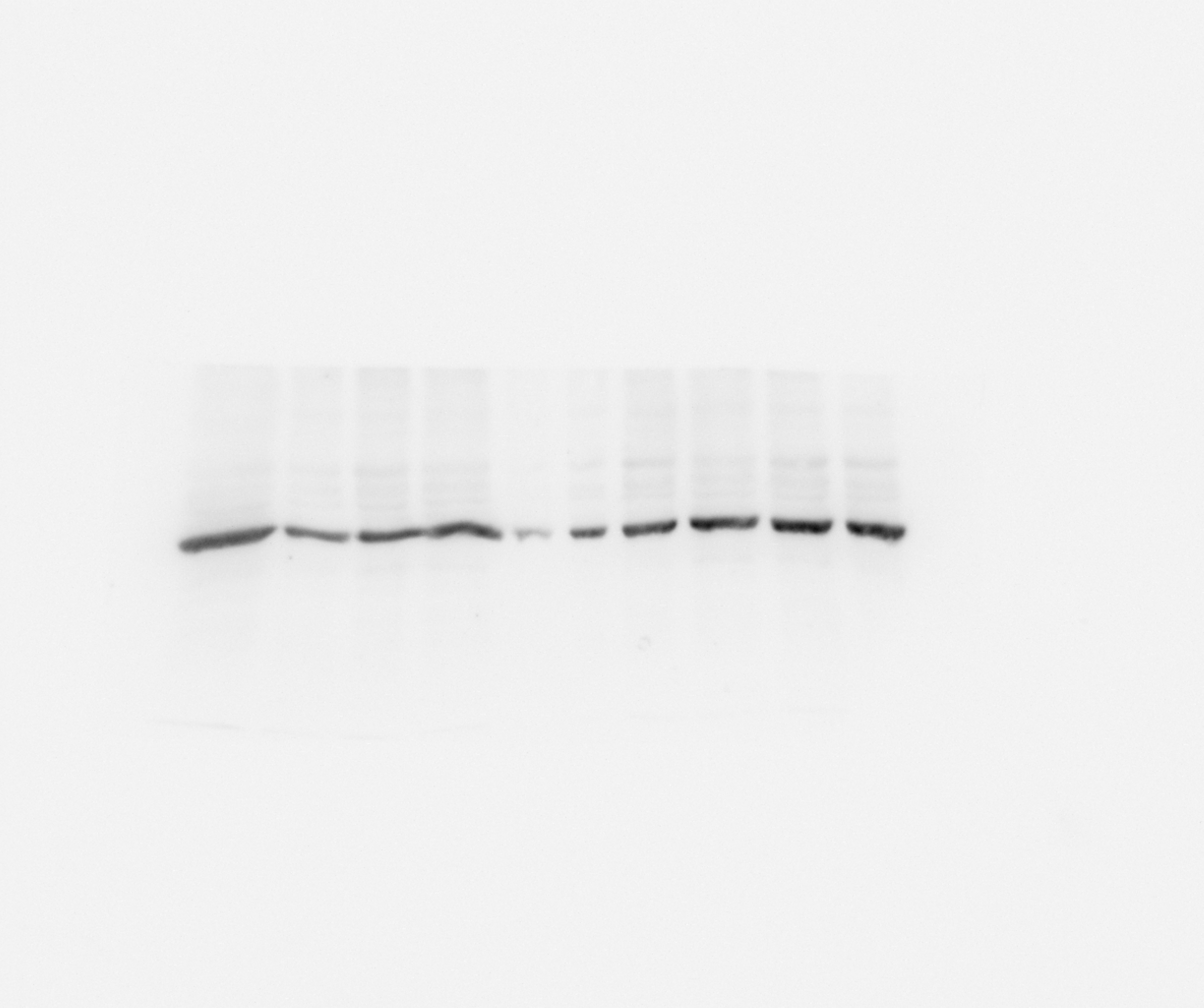

Supplement: Supplementary file 1 [file ijms-23-14638-s001.zip › Figure S3.tif]

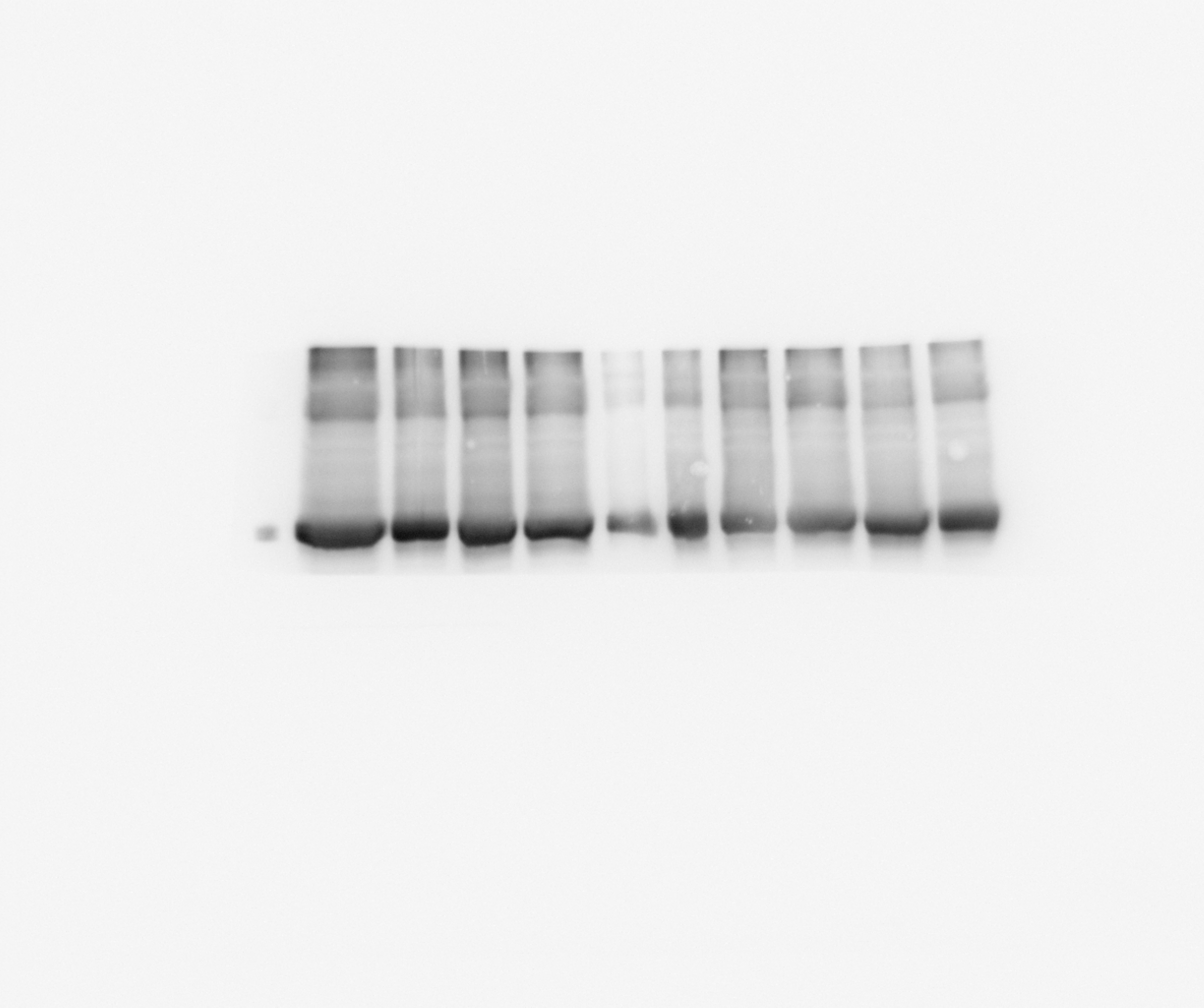

Supplement: Supplementary file 1 [file ijms-23-14638-s001.zip › Figure S4.tif]

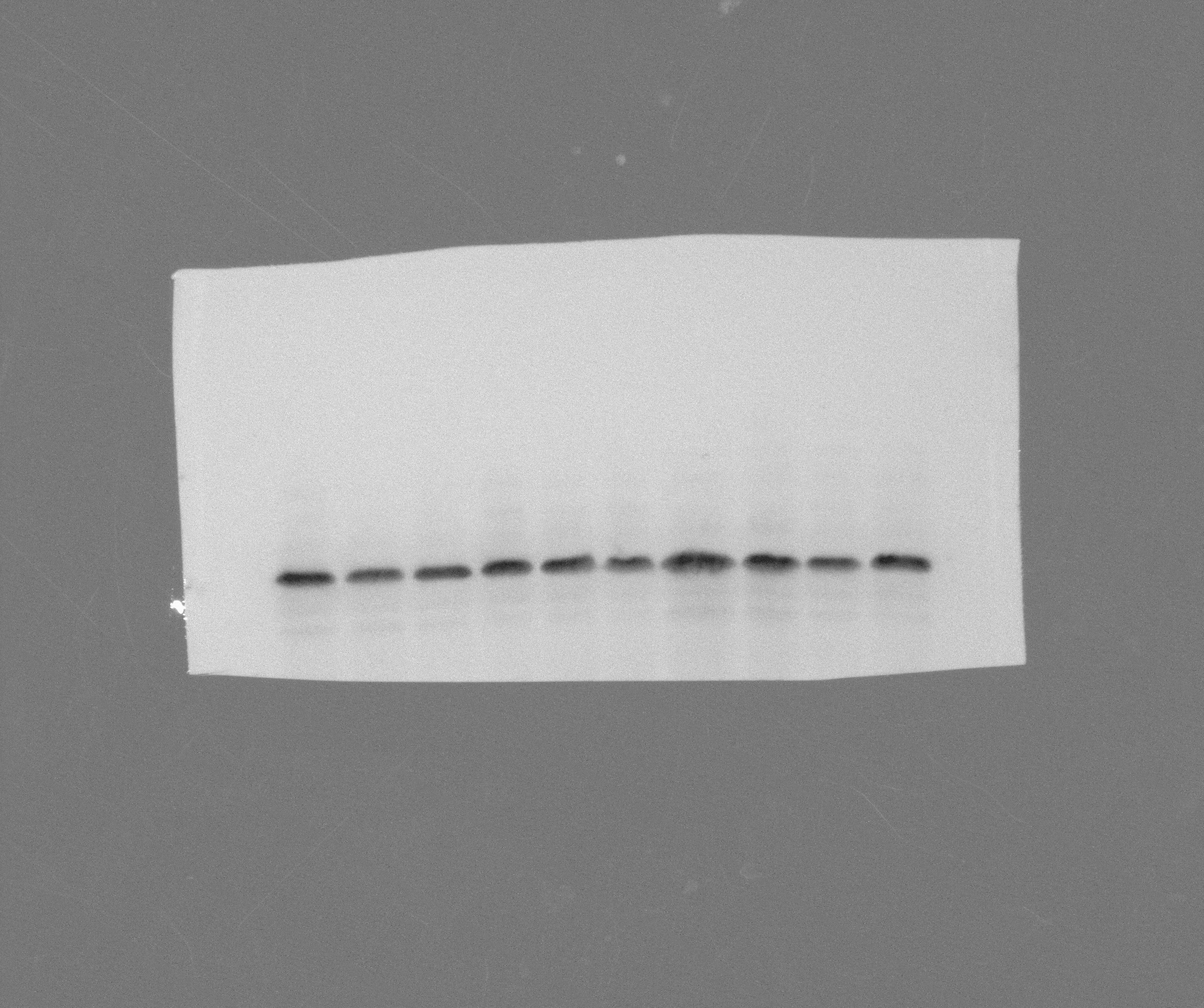

Supplement: Supplementary file 1 [file ijms-23-14638-s001.zip › Figure S5.tif]

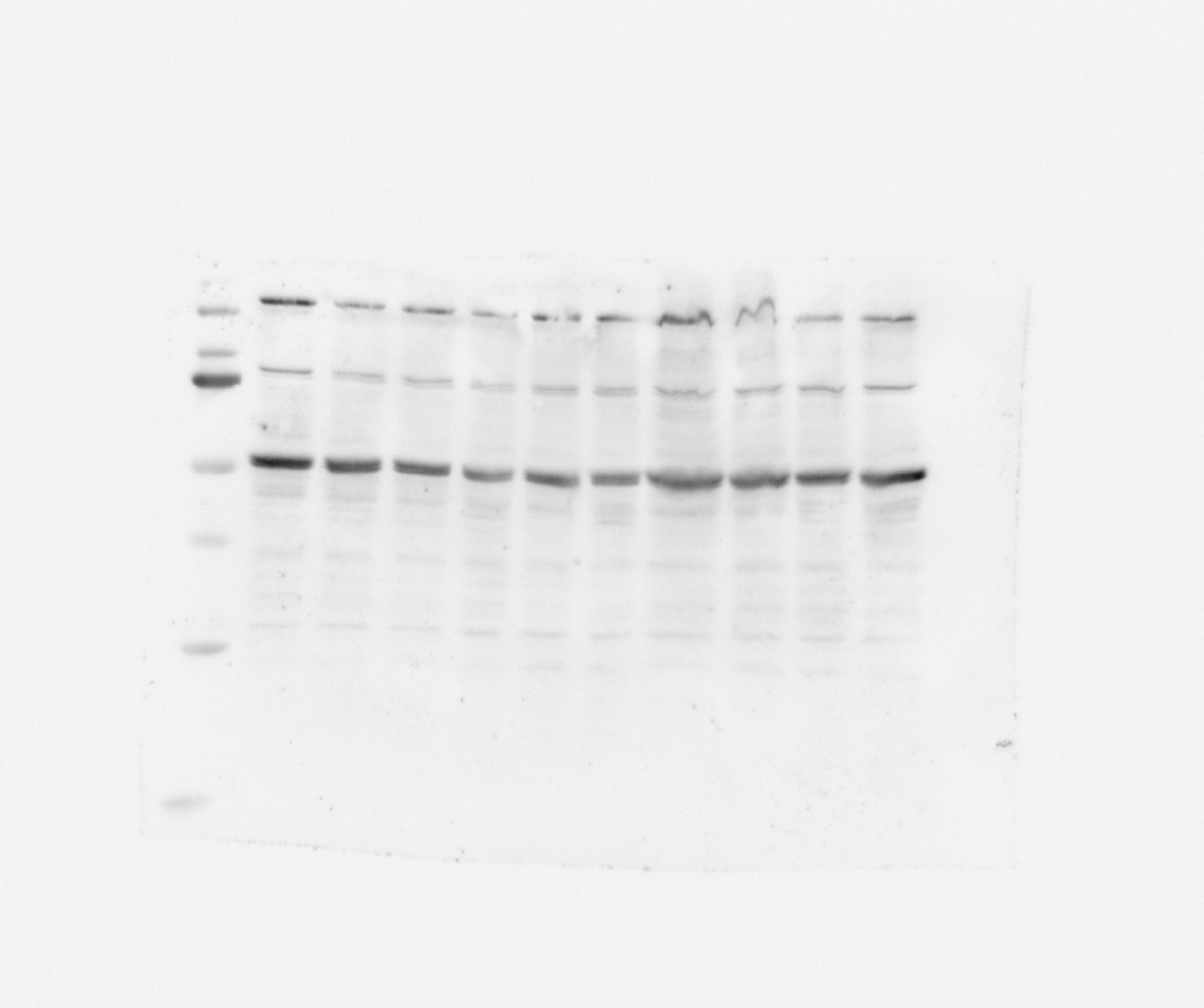

Supplement: Supplementary file 1 [file ijms-23-14638-s001.zip › Figure S6.tif]

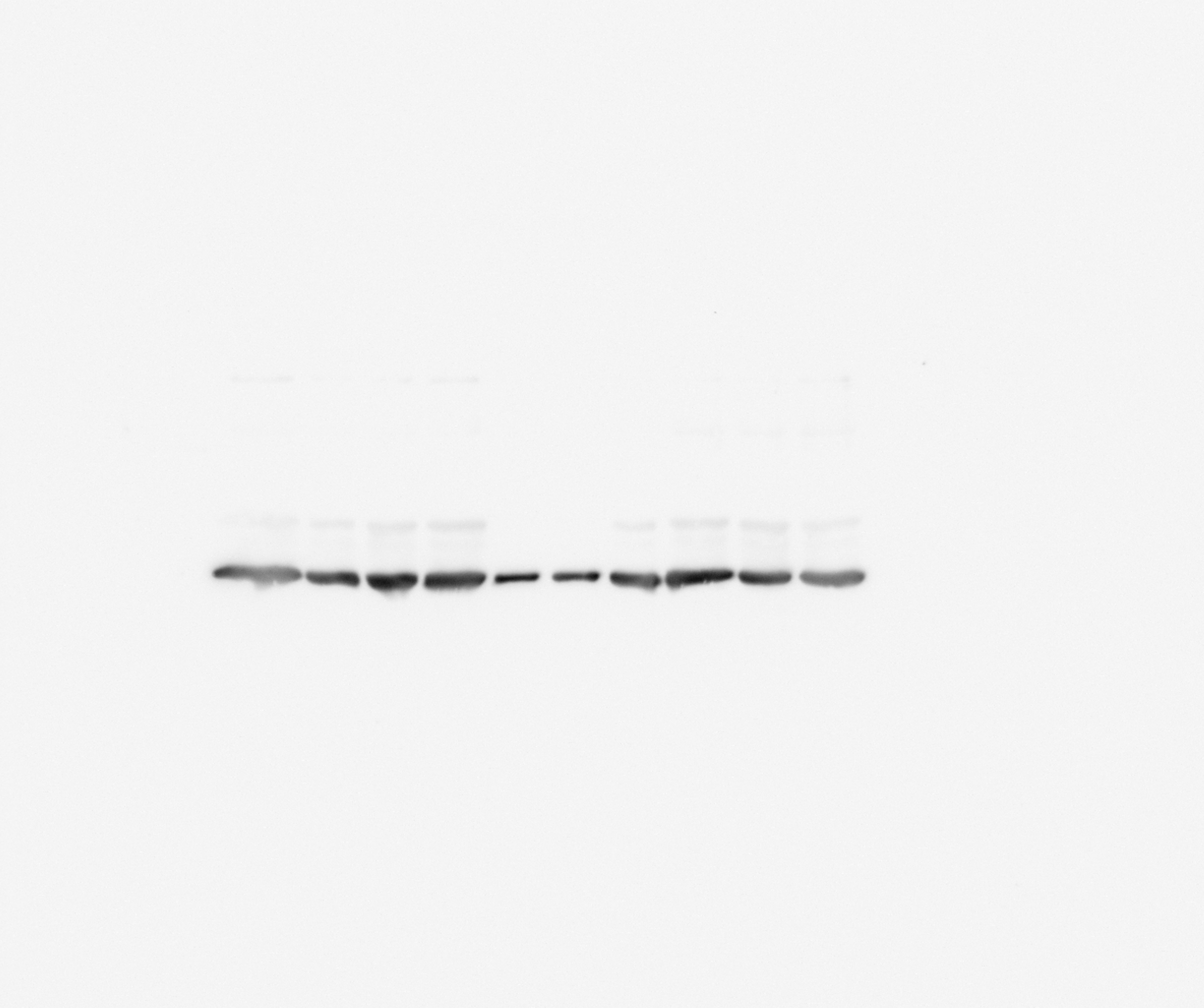

Supplement: Supplementary file 1 [file ijms-23-14638-s001.zip › Figure S7.tif]

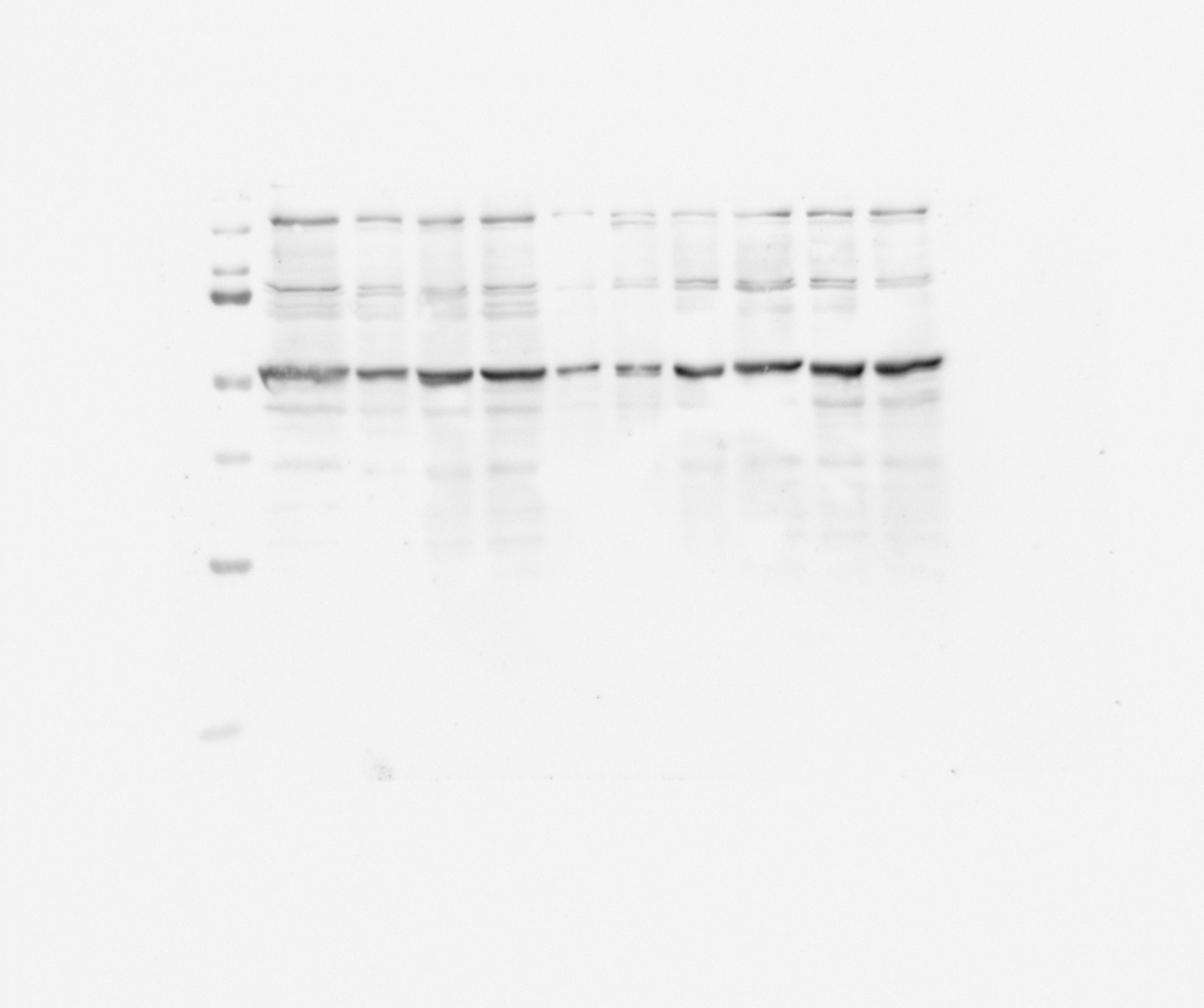

Supplement: Supplementary file 1 [file ijms-23-14638-s001.zip › Figure S8.tif]

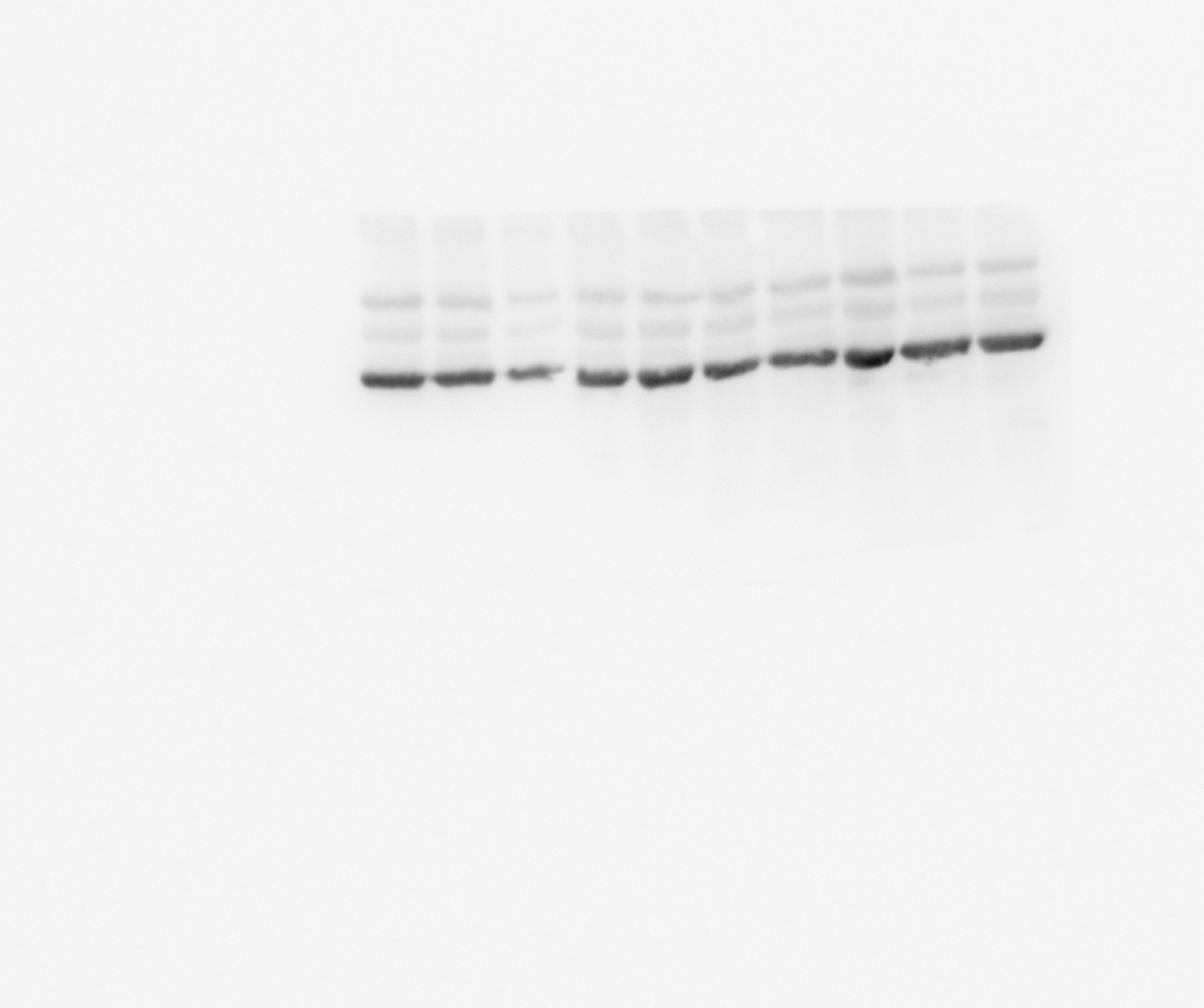

Supplement: Supplementary file 1 [file ijms-23-14638-s001.zip › Figure S9.tif]
